# Axisymmetric adaptive upper-bound finite element limit analysis formulation based on second-order cone programming for bearing capacity of circular footing

**DOI:** 10.1371/journal.pone.0321451

**Published:** 2025-06-05

**Authors:** Rui Sun, Haibing Cai, Kai Zhang

**Affiliations:** School of Civil Engineering and Architecture, Anhui University of Science and Technology, Huainan, Anhui, China; Universita degli Studi di Napoli Federico II, ITALY

## Abstract

An adaptive axisymmetric upper bound finite element limit analysis (UB-FELA) formulation has been presented for Mohr-Coulomb (M-C) materials in this paper. The computational domain is discretized using quadratic velocity elements. For the sake of computational efficiency, the axisymmetric UB finite element problem is recast into a model of second-order cone programming (SOCP). To enhance the precision of the proposed UB finite element method using a reduced element count, this study implements a mesh adaptation algorithm grounded in plastic dissipation. The collapse loads for determining the circular footings are then estimated by application of the proposed axisymmetric UB limit analysis formulas. By comparing the results to those reported in the literature, the analysis indicates that the method presented in this paper yields an accurate UB solution.

## 1 Introduction

The efficacy of the finite element limit analysis (FELA) method in solving geotechnical stability problems has been validated by numerous researchers. According to different situations, the problems can be classified into plane strain problems, axisymmetric and 3D problems. In practical engineering, the circular excavation and bearing capacity of circular foundation can be considered as axisymmetric problems.

Pastor and Turgeman [1] initially proposed an axisymmetric UB-FELA method, grounded in linear optimization techniques, to examine this problem. This method need enforce a total of 3*p* inequality constraints at each node, where *p* represents the number of sides of the polygon employed for the linearization of the yield surface. Nevertheless, the methodology presented by Pastor and Turgeman [1] appears to be computationally less efficient. Kumar and Khatri [2] and Kumar and Chakraborty [3] proposed a novel linearization method for the yield criterion that reduces computational complexity by requiring each node to be subject to only three additional inequality constraints. However, the substitution of the nonlinear yield function with a multitude of linear constraints results in an increase in computational cost that is prohibitive for large-scale problems. Recently, SOCP has been utilised by numerous researchers as a means of addressing significant optimisation challenges [4–9]. The formulation with the usage of SOCP or Semidefinite programming (SDP) permits the treatment of the yield function in its intrinsic form, this approach addresses the limitation of the linearisation of the yield function inherent to the linear programming technique. SOCP has been used in 2D M-C problems [4–7], axisymmetric M-C problems [8–9], and 3D Drucker-Prager (D-P) problems [10], where has shown the high computational efficiency in FELA method. Tang et al. [8] were the initial researchers to apply SOCP to the axisymmetric LB problem for M-C materials, adopting the formulation proposed by Pastor and Turgeman [1].The computational results reported from Tang et al. [8] have demonstrated that SOCP can effectively solve the lower bound problems than linear programming. Consequently, the application of SOCP is rendered feasible for enhancing the computational efficiency of the previously mentioned limit analysis method for M-C materials. This article provides detailed expressions for the flow rule constraints and element plastic dissipation for the axisymmetric UB limit analysis method based on M-C materials. This article presents a comprehensive account of the expressions utilized in the flow rule constraints and element plastic dissipation within the FELA method. The implementation of the axisymmetric UB-FELA method encompasses these key research areas.

On the other hand, many research results have shown that the use of mesh adaptive refinement strategy and high-order element in the UB-FELA method is an effective means to improve computational accuracy[6,7,10–12]. Makrodimopoulos and Martin [6] provided an UB-FELA method with 6-node high-order triangle elements for M-C Materials. Sun et al. [12] provided an UB-FELA method based on the quadratic velocity fields and mesh adaptive refinement strategy. However, the utilization of mesh adaptive refinement strategy and high-order element on the axisymmetric UB method still needs further research.

In this manuscript, a detailed theoretical basis for an axisymmetric upper limit finite element calculation method based on the SOCP technique and quadratic velocity element are presented. Additionally, A mesh adaptive algorithm driven by plastic dissipation is also proposed with the aim of obtaining highly accurate UB solutions. Numerous studies have confirmed the ability of the mesh adaptive strategy to improve the accuracy of plastic limit analysis calculations [5]. The study also reveals that the proposed method yields accurate UB solutions, as evidenced by a comparison with previous research findings on the stability of circular foundations involving both smooth and rough substrate-soil interfaces.

## 2 Axisymmetric UB-FELA with SOCP

### 2.1 Quadratic velocity finite element

**Fig 1:**
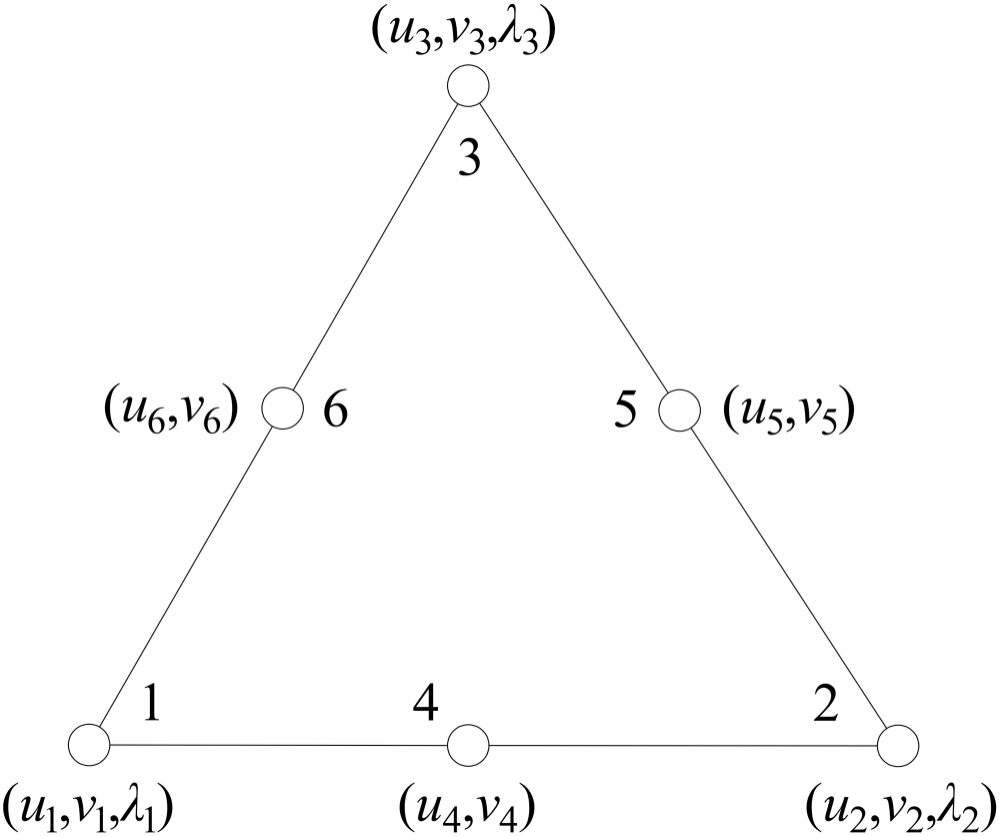
Quadratic velocity elements.


To discretize domain of computation, quadratic velocity elements are utilized. It has been demonstrated that quadratic velocity elements (6-node triangular element) can provide much better accuracy solutions but do not reduce the computation efficiency of solving UB optimization model [6]. Fig 1 shows the type of quadratic velocity element without velocities discontinuities between the adjacent elements. It is possible to express the velocity tensor at any specified point within a triangular element in terms of the velocity tensors at its six vertices.

$$\mathrm{\backslash [}u=\sum_{i=1}^{6}N_{i}u_{i},v=\sum_{i=1}^{6}N_{i}v_{i}\mathrm{\backslash ]}$$ (1)

In this context, *u*_*i*_ and *v*_*i*_ denote the velocities at a specified point in the radial (*r*) and axial (*z*) directions, respectively.

The term *N*_*i*_ represents the shape function associated with the quadratic velocity element and can be calculated using the following expressions.


$$\mathrm{\backslash [}N_{i}=\left(2L_{i}-1\right)L_{i}\left(i=1,2,3\right),N_{4}=4L_{1}L_{2},N_{5}=4L_{2}L_{3},N_{6}=4L_{3}L_{1}\mathrm{\backslash ]}$$ (2)

Where *L*_1_, *L*_2_, *L*_3_ is the shape function of the 3-nodes triangular element,


\[L1=(a1+b1r+c1z)/(a1+b1r+c1z)2A\nulldelimiterspace2AL2=(a2+b2r+c2z)/(a2+b2r+c2z)2A\nulldelimiterspace2AL3=(a3+b3r+c3z)/(a3+b3r+c3z)2A\nulldelimiterspace2A\] (3)

where,


$$\mathrm{\backslash [}\begin{array}{c} a_{1}=r_{2}z_{3}-r_{3}z_{2};b_{1}=z_{2}-z_{3};c_{1}=r_{3}-r_{2}\\ a_{2}=r_{3}z_{1}-r_{1}z_{3};b_{2}=z_{3}-z_{1};c_{2}=r_{1}-r_{3}\\ a_{3}=r_{1}z_{2}-r_{2}z_{1};b_{3}=z_{1}-z_{2};c_{3}=r_{2}-r_{1} \end{array}\mathrm{\backslash ]}$$ (4)

And $A=0.5\left|b_{1}c_{2}-b_{2}c_{1}\right|$ is the area of the element.

The necessary element strains for the axisymmetric problem can be determined through,


$$\mathrm{\backslash [}\varepsilon_{r}=\frac{\partial u}{\partial r},\varepsilon_{z}=\frac{\partial v}{\partial z},\gamma_{rz}=\frac{\partial u}{\partial z}+\frac{\partial v}{\partial r},\varepsilon_{\theta }=\frac{u}{r}\mathrm{\backslash ]}$$ (5)

In this context, the symbols *ε*_*r*_, *ε*_*z*_, and *ε*_*θ*_ are used to denote the plastic normal strain rates in the radial (*r*), axial (*z*), and circumferential (*θ*) directions, respectively. The symbol *γ*_*rz*_ is used to represent the plastic shear strain rate.

### 2.2 M-C Criterion for axisymmetric problem

The M-C Criterion in the context of an axisymmetric condition is formulated as follows [1,8].


$$\mathrm{\backslash [}\begin{array}{c} \sqrt{{\left(\sigma_{r}-\sigma_{z,i}\right)}^{2}+4\tau_{rz,i}^{2}}\leq 2c\cos\varphi +\left(\sigma_{r,i}+\sigma_{z,i}\right)\sin\varphi \\ \sqrt{{\left(\sigma_{r}-\sigma_{z,i}\right)}^{2}+4\tau_{rz,i}^{2}}\leq \frac{4c\cos\varphi }{1+\sin\varphi }+\left(\sigma_{r,i}+\sigma_{z,i}\right)-2\sigma_{\theta ,i}\frac{1-\sin\varphi }{1+\sin\varphi }\\ \sqrt{{\left(\sigma_{r}-\sigma_{z,i}\right)}^{2}+4\tau_{rz,i}^{2}}\leq \frac{4c\cos\varphi }{1-\sin\varphi }-\left(\sigma_{r,i}+\sigma_{z,i}\right)+2\sigma_{\theta ,i}\frac{1+\sin\varphi }{1-\sin\varphi } \end{array}\mathrm{\backslash ]}$$ (6)

The preceding three criteria can also be expressed in the form of three distinct types of 3D second-order cones.


$$\mathrm{\backslash [}\begin{array}{c} A_{1}\sigma +b_{1}\in K_{1}^{*}\\ A_{2}\sigma +b_{2}\in K_{2}^{*}\\ A_{3}\sigma +b_{3}\in K_{3}^{*} \end{array}\mathrm{\backslash ]}$$ (7)

Where $\sigma =\left(\sigma_{r},\sigma_{z},\tau_{rz},\sigma_{\theta }\right)$, $\sigma_{r},\sigma_{z},\tau_{rz},\sigma_{\theta }$ is nodal stresses, $K_{i}^{*}$ represents second-order cone constraint.


A1=[−sinφ−sinφ    0    0  −1      1        0    0     0       0    −2    0]A2=[ −1 −1   0  2  1−sinφ/(1−sinφ)(1+sinφ)\nulldelimiterspace1+sinφ −1    1    0               0   0    0 −2               0]A3=[   1    1     0  −2(1+sinφ)/(1+sinφ)(1−sinφ)\nulldelimiterspace(1−sinφ) −1    1     0                 0   0    0  −2                 0] (8a)

$$\mathrm{\backslash [}\begin{array}{c} b_{1}={\left\{2c\cos\varphi 00\right\}}^{T}\\ b_{2}={\left\{\frac{4c\cos\varphi }{1+\sin\varphi }00\right\}}^{T}\\ b_{3}={\left\{\frac{4c\cos\varphi }{1-\sin\varphi }00\right\}}^{T} \end{array}\mathrm{\backslash ]}$$ (8b)

### 2.3 The plastic strain dissipation function and associated flow rule constraints

This section comprehensively introduces the application of SOCP within the axisymmetric UB-FELA methodology for M-C materials. The initial requirement for employing the UB limit analysis method is to derive an expression for the dissipation function, as well as the delineation of the set of plastically admissible strains[13]. Under axisymmetric conditions, the M-C criterion can be reformulated as a SOCP, leveraging the duality principles inherent in conic optimization based on Eq. (6).

In fact, it is difficult to directly establish the associated flow rule constraints without stress components based on axisymmetric M-C Criterion conditions. According to the research results of Makrodimopoulos [14], the analytical expressions for the associated flow rule constraints and plastic dissipation can be obtained by solving Eq. (9a).


$$\mathrm{\backslash [}\begin{array}{c} \sup\varepsilon^{T}\sigma \\ s.t.A_{i}\sigma +b_{i}\in K_{i}^{*}i=1,2,3 \end{array}\mathrm{\backslash ]}$$ (9a)

where $\sigma =\left(\sigma_{r},\sigma_{z},\tau_{rz},\sigma_{\theta }\right)$, $\varepsilon =\left(\varepsilon_{r},\varepsilon_{z},\varepsilon_{rz},\varepsilon_{\theta }\right)$. By solving Eq. (9a), the analytical expressions for the associated flow rule constraints and plastic dissipation shown in Eq. (9b) can be obtained.


$$\mathrm{\backslash [}\begin{array}{c} \inf\sum_{i=1}^{n}b_{i}^{T}y_{i}\\ s.t.-\sum_{i=1}^{n}A_{i}^{T}y_{i}=\varepsilon \\ y_{i}\in K_{i}^{*},i=1,...,n \end{array}\mathrm{\backslash ]}$$ (9b)

where $A_{i}^{T}\left(i=1,2,3\right)$ can be expressed by Eq.(10).


$$\begin{array}{c} A_{1}^{T}=\left[\begin{array}{c} -\sin\varphi \;-1\;0\\ -\sin\varphi \;1\;0\\ \;0\;0\;-2\\ \;0\;0\;0 \end{array}\right] \end{array}$$ (10a)

$$\begin{array}{c} A_{2}^{T}=\left[\begin{array}{c} -1\;-1\;0\\ -1\;1\;0\\ \;0\;0\;-2\\ 2\frac{1-\sin\varphi }{1+\sin\varphi }\;0\;0 \end{array}\right] \end{array}$$ (10b)

$$\begin{array}{c} A_{3}^{T}=\left[\begin{array}{c} \;1\;-1\;0\\ \;1\;1\;0\\ \;0\;0\;-2\\ -2\frac{1+\sin\varphi }{1-\sin\varphi }\;0\;0 \end{array}\right] \end{array}$$ (10c)

Utilizing this method, According to Eq. (8), Eq. (9b), and Eq. (10), the expression for the dissipation function is reformulated as shown in Eq. (11).


$$\mathrm{\backslash [}d_{p}=\sum_{i=1}^{n}b_{i}^{T}y_{i}=2c\cos\varphi y_{1}^{1}+\frac{4c\cos\varphi }{1+\sin\varphi }y_{2}^{1}+\frac{4c\cos\varphi }{1-\sin\varphi }y_{3}^{1}\mathrm{\backslash ]}$$ (11)

Additionally, the associated flow rule constraints for each element can be obtained as follows,


$$\mathrm{\backslash [}\begin{array}{ccc} {}*20c\left\{ \begin{array}{c} -\sum_{i=1}^{n}A_{i}^{T}y_{i}=\varepsilon \\ y_{i}\in K_{i}^{*},i=1,...,n \end{array}\right. & \to & \left\{ \begin{array}{c} \varepsilon_{r}+\left(-\sin\varphi y_{1}^{1}-y_{1}^{2}\right)+\left(-y_{2}^{1}-y_{2}^{2}\right)+\left(y_{3}^{1}-y_{3}^{2}\right)=0\\ \varepsilon_{z}+\left(-\sin\varphi y_{1}^{1}+y_{1}^{2}\right)+\left(-y_{2}^{1}+y_{2}^{2}\right)+\left(y_{3}^{1}+y_{3}^{2}\right)=0\\ \gamma_{rz}+\left(-2y_{1}^{3}\right)+\left(-2y_{2}^{3}\right)+\left(-2y_{3}^{3}\right)=0\\ \varepsilon_{\theta }+0+\left(2\frac{1-\sin\varphi }{1+\sin\varphi }y_{2}^{1}\right)+\left(-2\frac{1+\sin\varphi }{1-\sin\varphi }y_{3}^{1}\right)=0\\ y_{1}^{1}\geq \sqrt{{\left(y_{1}^{2}\right)}^{2}+{\left(y_{1}^{3}\right)}^{2}}\\ y_{2}^{1}\geq \sqrt{{\left(y_{2}^{2}\right)}^{2}+{\left(y_{2}^{3}\right)}^{2}}\\ y_{3}^{1}\geq \sqrt{{\left(y_{3}^{2}\right)}^{2}+{\left(y_{3}^{3}\right)}^{2}} \end{array}\right. \end{array}\mathrm{\backslash ]}$$ (12)

Where $y_{i}={\left(y_{i}^{1},y_{i}^{2},y_{i}^{3}\right)}^{T},i=1,2,3$ are the auxiliary variables, $\varepsilon_{r},\varepsilon_{z},\gamma_{rz},\varepsilon_{\theta }$ are strain variables.

## 3 Numerical formulation

### 3.1 Calculation of power dissipation.

To mitigate the likelihood of errors during the computational process, the current section initially provides an analytical expression that describes the rate of external work related to body forces and the dissipation of power resulting from plastic deformation within the framework of continuous quadratic velocity fields. The further details regarding the derivation of the formula are presented in Appendix. In an axisymmetric problem solved by means of SOCP optimization, the power dissipated due to plastic deformation can be expressed through the subsequent formula:


$$\mathrm{\backslash [}P_{e}=\int_{A}d_{p}dA\mathrm{\backslash ]}$$ (13)

where the rate of external work performed by body forces is given by the following analytical expression:


$$\mathrm{\backslash [}P_{t}=\int_{A}\gamma vdA\mathrm{\backslash ]}$$ (14)

In this expression, *A* signifies the area of the element, *γ* denotes the unit weight of the soil mass, *v*_*i*_ corresponds to the vertical velocity at the node, and *r*_1_, *r*_2_, *r*_3_ represent the radial coordinates for the three vertices of the triangular element.

### 3.2 Numerical implementation.

For a six-node triangular element, the plastic strain rate field (*ε*_*r*_*, ε*_*z*_*, ε*_*rz*_) exhibits linear variation, whereas *ε*_*θ*_ remains a constant, corresponding to the strain at the centroid of the triangular element. The quadratic velocity fields is applied in the UB limit analysis method to help obtain high-precision UB solution. The flow rule constraint for axisymmetric UB formulation can be applied in the three vertices. This paper formulates the axisymmetric UB problem, incorporating finite elements, as a conventional SOCP problem. In fact, the large scale mathematical optimization calculation model based on second-order cone programming technology has been widely proven to be efficiently solved[15–20].Constructing an upper bound finite element limit analysis calculation model that satisfies the associated flow rule and velocity boundary conditions everywhere can ensure obtaining strict upper bound solutions. The axisymmetric upper bound finite element method proposed in this article assumes that the circumferential strain *ε*_*θ*_ inside each element is a constant. This assumption means that Eq. (15) does not fully comply with the associated flow rule, and this present method cannot obtain a strict upper bound solution. However, based on existing research [3,11] and the results of this study, this assumption does not lead to computational errors.


$$\begin{array}{c} \begin{array}{cc} & \min\;\sum_{k=1}^{NE}P_{e,k}-\sum_{k=1}^{NE}P_{t,k}\\ & \text{Subject to}\\ & \left\{ \begin{array}{cc} & \;\;\;\;\;\;\;\varepsilon_{r,i}+\left(-\sin\phi y_{1,i}^{1}-y_{1,i}^{2}\right)+\left(-y_{2,i}^{1}-y_{2,i}^{2}\right)+\left(y_{3,i}^{1}-y_{3,i}^{2}\right)=0\;(i=1,...,NP)\\ & \;\;\;\;\;\;\;\varepsilon_{z,i}+\left(-\sin\phi y_{1,i}^{1}+y_{1,i}^{2}\right)+\left(-y_{2,i}^{1}+y_{2,i}^{2}\right)+\left(y_{3,i}^{1}+y_{3,i}^{2}\right)=0\;(i=1,...,NP)\\ & \;\;\;\;\;\;\;\gamma_{rz,i}+\left(-2y_{1,i}^{3}\right)+\left(-2y_{2,i}^{3}\right)+\left(-2y_{3,i}^{3}\right)=0\;(i=1,...,NP)\\ & \;\;\;\;\;\;\;\varepsilon_{\theta ,i}+0+\left(2\frac{1-\sin\varphi }{1+\sin\varphi }y_{2,i}^{1}\right)+\left(-2\frac{1+\sin\varphi }{1-\sin\varphi }y_{3,i}^{1}\right)=0\;(i=1,...,NP)\\ & \;\;\;\;\;\;\;y_{1,i}^{1}\geq \sqrt{{\left(y_{1,i}^{2}\right)}^{2}+{\left(y_{1,i}^{3}\right)}^{2}}\;(i=1,...,NP)\\ & \;\;\;\;\;\;\;y_{2,i}^{1}\geq \sqrt{{\left(y_{2,i}^{2}\right)}^{2}+{\left(y_{2,i}^{3}\right)}^{2}}\;(i=1,...,NP)\\ & \;\;\;\;\;\;\;y_{2,i}^{1}\geq \sqrt{{\left(y_{3,i}^{2}\right)}^{2}+{\left(y_{3,i}^{3}\right)}^{2}}\;(i=1,...,NP)\\ & \;\;\;\;\;\;\;\varepsilon_{r,i}=\frac{\partial u}{\partial r},\;\varepsilon_{z,i}=\frac{\partial v}{\partial z},\;\gamma_{rz,i}=\frac{\partial u}{\partial z}+\frac{\partial v}{\partial r},\;\varepsilon_{\theta ,i}=\frac{u}{r}\;(i=1,...,NP)\\ & \;\;\;\;\;\;\;u_{i}\cos\omega_{i}+v_{i}\sin\omega_{i}={\bar{u}}_{i}\;(i=1,...,NB)\\ & \;\;\;\;\;\;\;v_{i}\cos\omega_{i}-u_{i}\sin\omega_{i}={\bar{v}}_{i}\;(i=1,...,NB) \end{array}\right. \end{array} \end{array}$$ (15)

In which *NE* denotes the element number, *NP* represents the flow rule point number, *NB* is the boundary point number, and *NP* = 3*NE.* The strain components ε_r_, ε_z_, γ_rz_, ε_θ_ can be converted into the expression of velocity components based on Eq.(1) and Eq.(5). ω represents the angle between the velocity boundary and the horizontal coordinate axis. The analytical expression of flow rule constraints and plastic dissipation is of great significance for the constructing upper bound finite element limit analysis calculation programs. The upper bound finite element limit analysis model based on second-order cone programming can be obtained through Eq. (15), which is only related to node velocity variables and second-order cone optimization variables.

Moreover, the computational program for the UB finite element method, founded on SOCP and utilizing continuous secondary velocity field, is coded in MATLAB. All computations were executed on a Lenovo system equipped with a 3.2 GHz CPU and 8GB RAM, operating under the Windows 7 operating system.

## 4 Mesh adaptive strategy

Numerous scholars have adapted the adaptive meshing technique for application within the FELA method [11,21]. Nonetheless, research on the application of adaptive meshing techniques to axisymmetric problems remains nascent. Zhang et al. [21] introduced an adaptive meshing strategy based on plastic dissipation for axisymmetric UB limit analysis problems. This paper presents a plastic-dissipation-based adaptive strategy to enhance the precision of determining UB solutions and associated failure mechanisms. The proposed plastic-dissipation-based adaptive meshing strategy is analogous to that introduced by Zhang et al. [21], employing a 3-node triangular element. The mesh adjacent to failure zones should be refined, whereas the mesh located farther from plasticized zones may be coarsened incrementally. It appears that elements adjacent to the failure regions are likely to exhibit high plastic-dissipation *P*_*e*_ values. The plastic-dissipation *P*_*e*_ values for each element are sorted in descending order. Consequently, elements requiring refinement can be identified using the local refinement indicator *η*.


\[η=∑i=1nePe,i/∑i=1nePe,i∑i=1NEPe,i\nulldelimiterspace∑i=1NEPe,i(0≤η≤1)\] (16)

In which *n*_*e*_ denotes the total count of marked elements, *NE* represents the total count of all elements. An appropriate value for *η* can be determined through trial calculations, typically lying within the range of 0.4 to 0.5. A value of *η* equal to 0 signifies that no elements should be refined, whereas *η* equal to 1 indicates that all elements within the computational domain are to undergo a refinement process. The adaptive limit analysis program is implemented following the methodology outlined below.

1. Address the UB problem as a SOCP optimized method, which based on using the initial set of elements.
2. Initially, the elements are sorted in descending order based on the computed plastic damage value *P*_*e*_, followed by the identification of elements for refinement based on the refinement factor *η*. The plastic-dissipation *P*_e_ values for each element can be obtained based on Eq. (21), in which the necessary data can be extracted from the solution results.
3. Two new subtriangles are generated from each parent triangle by connecting the vertices of the parent triangle to the midpoint of its longest side.
4. Based on the refined mesh data, rebuild the upper bound finite element limit analysis calculation model. Return to step 1 and start a new calculation.
5. Each adaptive step is iterated through the predetermined number of iterations to achieve the optimal UB solution and the associated failure mechanism.

## 5 Numerical examples

The bearing capacity of circular foundations is a classic axisymmetric stability problem[21–26]. A procedure was conducted to compute the bearing capacity of circular foundations subjected to both smooth and rough substrate-soil interfaces, and the accuracy and efficacy of this procedure were evaluated based on the resultant outcomes. The accuracy, computational efficiency, and failure mechanisms associated with the structures were assessed in the context of the UB solution.

**Fig 2:**
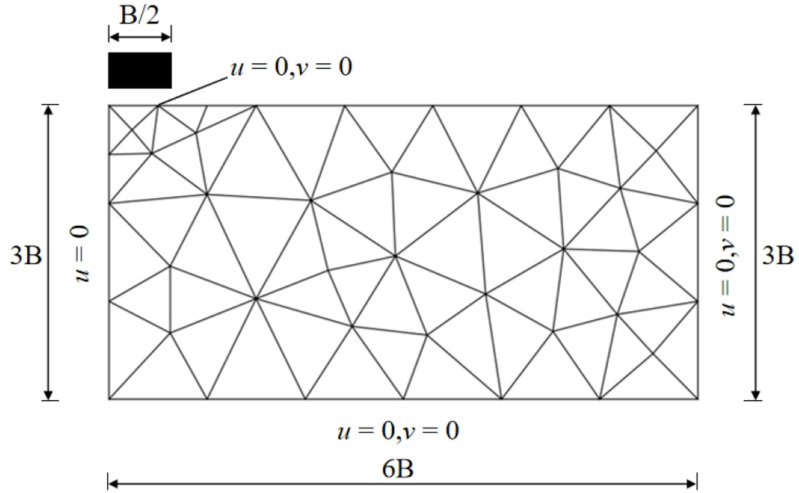
General layout and typical mesh of the problem (rough interface) for UB method.

Only half of the total construction shown in Fig.2 is considered and B is diameter of circular footing. The soil mass is characterized as a M-C material, possessing soil unit weight (*γ*), soil cohesion (*c*), and internal friction angle (*φ*).

The bearing capacity for a rigid circular footing devoid of surcharge is expressed in [8] as:


\[cNc=QuπB2/πB24\nulldelimiterspace4\] (17)

Wherein *N*_*c*_, which is a function of the internal friction angle *φ*, denotes the coefficient of load-bearing capacity. *Q*_u_ is the ultimate bearing capacity of circular foundation, which can be obtained by the upper bound model shown as Eq. (15). The perfectly rough footing-soil interface condition can be simulated by specifying the node velocity in the *u* and *v* directions for the footing nodes, *u* = 0, *v* = -1. For a smooth footing, the interface condition can be simulated by specifying the node velocity in the *v* direction for the footing nodes, *v* = -1.

### 5.1 Method validation

Table 1 presents the bearing capacity coefficients *N*_*c*_ for both smooth (bracketed) and rough (unbracketed) substrate-soil interfaces, with friction angles *φ* ranging from 5° to 30°. The results are juxtaposed with (b) the UB solutions of Mohapatra and Kumar [11], which employ discontinuous quadratic velocity fields, (c) the UB solutions of Kumar and Chakraborty [3], utilizing a three-node element with velocity discontinuity, (d) the UB solutions of Zhang et al. [21], which are based on a three-node element with velocity discontinuity and employ a mesh adaptive strategy, (e) the LB solutions of Kumar and Khatri [2], obtained using a three-node element, (f) solutions derived from the stress characteristics method [23], (g) solutions derived from the stress characteristics method [22], (h) numerical solutions based on the FLAC 2D method [24]. Table 1 indicates that the results derived from the current analysis are in reasonable agreement with the various solutions documented in the literature. It is significant to observe that the bearing capacity values for the smooth footing-soil interface are positioned between the UB and LB solutions as documented in the literature. This implies that the current method is capable of yielding UB solutions with satisfactory accuracy. Additionally, it is observed that the UB solution obtained from the present method is marginally higher than that derived from the stress characteristics method.

**Table 1:** Comparison between the *N*_*c*_ values of the circular footings.

*φ*(°)	Present method	Mohapatra [11]	Kumar [3]	Zhang [21]	Kumar [11]	Martin [23]	Cox [22]	Erickson [24]
5	8.08(7.45)	8.12(7.60)	8.11(7.58)	8.00(7.47)	8.06(7.31)	8.06(7.43)	(7.44)	–
10	11.12(10.01)	11.07(10.18)	11.18(10.19)	11.16(10.05)	10.99(9.78)	11.09(9.99)	(9.98)	–
15	15.91(13.92)	15.83(14.16)	16.10(14.19)	16.03(13.99)	15.66(13.51)	15.84(13.87)	(13.9)	–
20	23.80(20.14)	23.68(20.31)	24.24(20.65)	24.02(20.32)	23.22(19.38)	23.67(20.07)	(20.1)	22.30(19.50)
25	37.65(30.61)	37.36(31.00)	38.62(31.68)	37.96(30.97)	36.17(29.06)	37.31(30.52)	(30.5)	–
30	63.01(49.45)	62.93(50.14)	65.65(51.77)	–	61.48(47.10)	62.70(49.29)	(49.3)	–

Note: Values outside parentheses denote rough footing-soil interfaces, whereas those inside denote smooth interfaces.

### 5.2 Discussion on the effectiveness of mesh adaptive strategy

The computation accuracy can be also improved by using more elements, but it seems to be a large time consumption. It has been proved that an adaptive refinement method can help obtain high-precision results with fewer elements in axisymmetric UB problems [21]. A plastic dissipation-driven mesh adaptation strategy, akin to the approach presented in literature [21], is employed in this paper to enhance the UB solutions.

**Fig 3:**
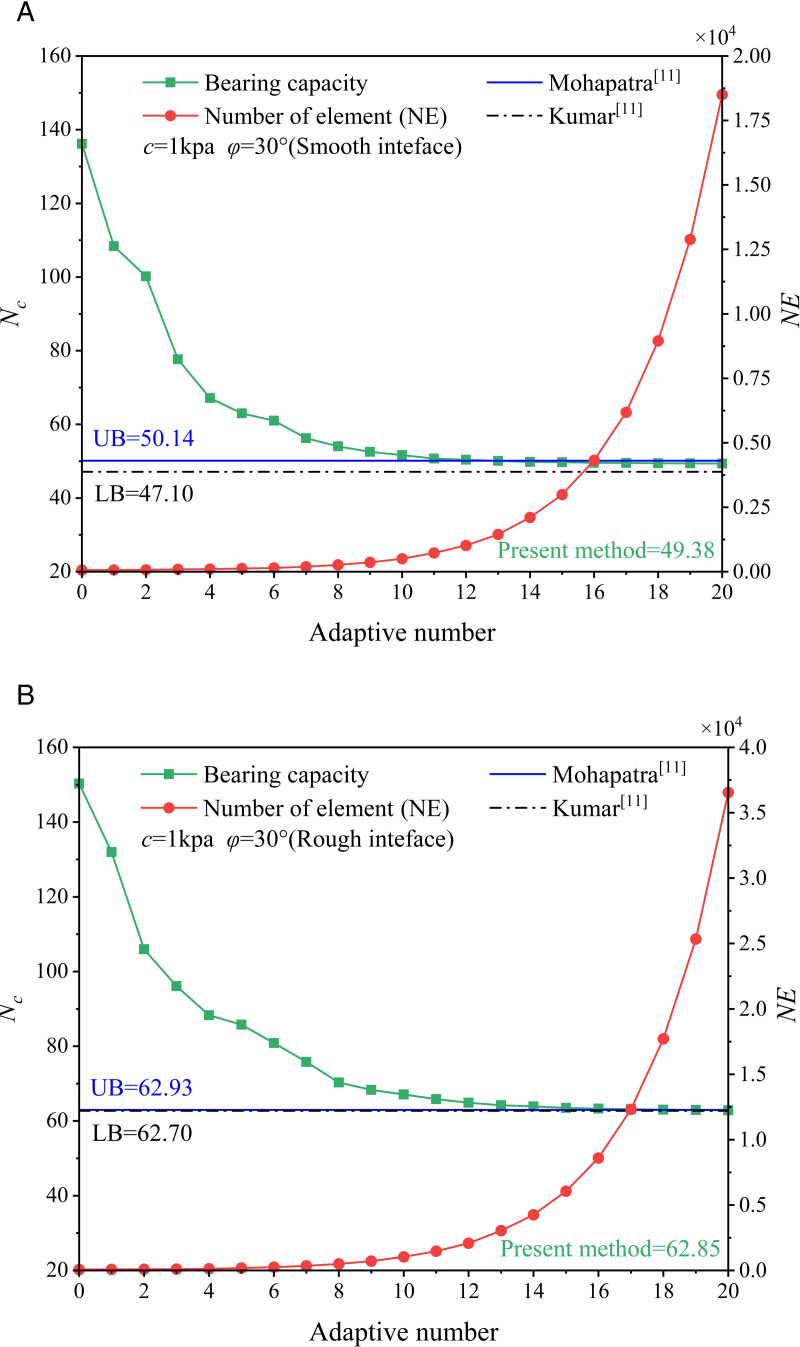
The impact of the number of iterations on the load carrying capacity factor and the element count.

Fig 3 indicates that the adaptive strategy adopted by this paper can improve the calculation accuracy of UB solution. However, note that the element number increases gradually with the adaptive number increases, but the calculation accuracy is limitedly improved after a few times mesh refinement. It means that if the numeric results are approximate to actual value, improving the computational results by using mesh refinement becomes very difficult.

**Fig 4:**
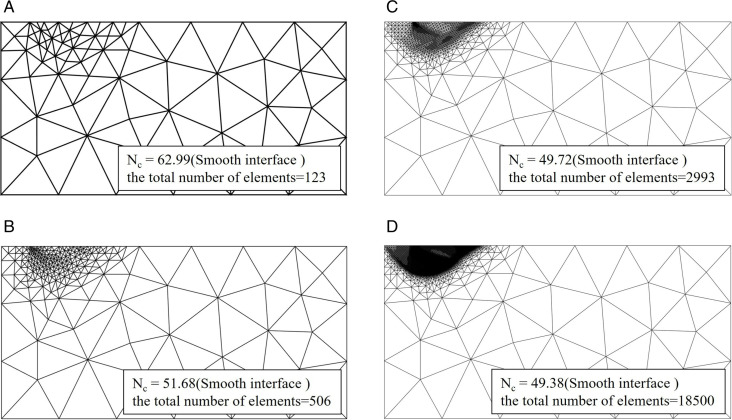
Mesh refinement in the calculation of *N*_*c*_ for smooth interface with *φ* = 30°. (a) fifth adaptive mesh refinement. (b) tenth adaptive mesh refinement. (c) fifteenth adaptive mesh refinement. (d) twentieth adaptive mesh refinement.

**Fig 5:**
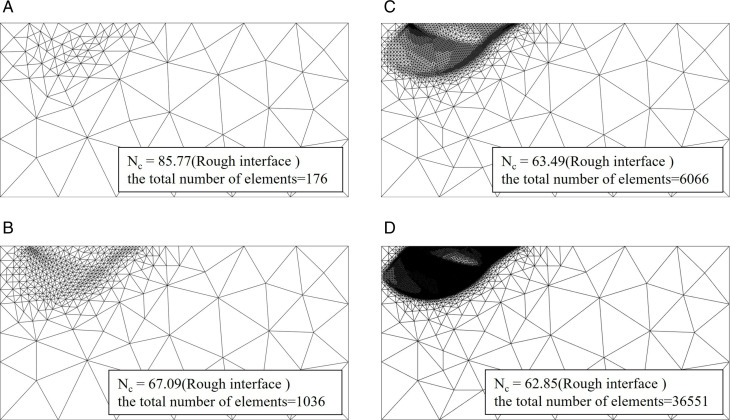
Mesh refinement in the calculation of *N*_*c*_ for rough interface with *φ* = 30°. (a) fifth adaptive mesh refinement. (b) tenth adaptive mesh refinement. (c) fifteenth adaptive mesh refinement. (d) twentieth adaptive mesh refinement.

Fig 4 and Fig 5 illustrates the alterations in the refined mesh and the bearing capacity coefficient *N*_*c*_ for both smooth and rough foundation-soil interfaces at a friction angle of *φ* = 30°. It is important to note that only the local region is depicted to facilitate a clearer explanation of the issue. The area of damage associated with the rough footing is markedly greater than that associated with the smooth footing, and a distinct disparity exists between the damage modes of the two footings. The observed failure pattern is in close agreement with the characteristic meshes presented by Martin [23]. Fig 4 and Fig 5 further indicates that the arrangement of the refined mesh parallels the patterns of power dissipation, consequently, the refined mesh configuration proposed herein may indirectly reveal the failure pattern. Additionally, Fig 4 and Fig 5 demonstrates that the meshes located distant from the failure zone are significantly coarser compared to those within the failure zone, this inference suggests why the adaptive refinement strategy is effective in achieving a superior UB solution with a reduced number of elements.

### 5.3 Detailed Nc values for the rigid circular footing

The precise UB and LB solutions for the bearing capacity coefficients *N*_*c*_ of smooth (bracketed) and rough (unbracketed) substrate-soil interfaces are presented in Table 2, with the friction angle *φ* ranging from 0° to 39°. The UB solutions listed in Table 2 are derived from the presented UB method, whereas the LB solutions are obtained through the LB method proposed by Sun and Yang [27]. It should be noted that the UB and LB solutions for bearing capacity, corresponding to smooth and rough footing-soil interfaces, are closely aligned. This implies that the current method is capable of yielding UB solutions with satisfactory accuracy.

**Table 2:** The UB and LB of the bearing capacity factors *N*_c._

*φ*	Rough interface		Smooth interface		*φ*	Rough interface		Smooth interface	
	UB	LB	UB	LB		UB	LB	UB	LB
0	6.07	6.04	5.70	5.68	20	23.80	23.60	20.14	20.03
1	6.41	6.38	6.00	5.98	21	25.95	25.71	21.80	21.68
2	6.78	6.75	6.33	6.31	22	28.37	28.09	23.67	23.50
3	7.18	7.15	6.68	6.65	23	31.08	30.77	25.72	25.56
4	7.61	7.58	7.05	7.02	24	34.20	33.75	28.05	27.84
5	8.08	8.05	7.45	7.42	25	37.65	37.15	30.61	30.42
6	8.59	8.56	7.88	7.86	26	41.56	40.96	33.53	33.27
7	9.15	9.11	8.36	8.32	27	46.00	45.31	36.79	36.51
8	9.75	9.71	8.86	8.83	28	51.03	50.24	40.47	40.17
9	10.41	10.36	9.42	9.38	29	56.81	55.86	44.71	44.32
10	11.12	11.07	10.01	9.97	30	63.01	62.28	49.45	49.04
11	11.91	11.85	10.66	10.62	31	71.26	69.68	54.95	54.40
12	12.77	12.70	11.37	11.32	32	80.02	78.19	61.24	60.55
13	13.72	13.64	12.14	12.09	33	90.22	88.00	68.36	67.66
14	14.77	14.67	12.98	12.93	34	102.06	99.48	76.76	75.77
15	15.91	15.81	13.92	13.85	35	116.78	112.79	86.34	85.23
16	17.19	17.06	14.93	14.86	36	133.19	128.05	97.70	96.26
17	18.59	18.45	16.04	15.96	37	152.53	146.64	110.76	109.07
18	20.15	19.99	17.27	17.19	38	175.79	167.47	126.24	124.17
19	21.87	21.69	18.63	18.53	39	202.79	192.52	144.54	141.91

## 6 Conclusions

An upper bound limit analysis (UB-LA) formulation has been proposed to address axisymmetric geomechanically stability challenges, integrating finite element method and SOCP optimization techniques. This study also incorporates a mesh adaptive refinement strategy and employs high-order elements to facilitate the acquisition of high-precision UB solutions with a reduced number of elements. The results of the current analysis are demonstrated to be in close alignment with the UB and LB solutions documented in the literature. Consequently, the developed method is employed to examine the bearing capacity of a circular foundation in relation to both smooth and rough footing-soil interfaces. The method holds considerable promise as a technique for deriving highly precise UB solutions and delineating distinct failure patterns in axisymmetric geomechanically scenarios.

## Appendix

The rate at which external work is performed by body forces is defined as,


$$\mathrm{\backslash [}P_{t}=\int_{A}\gamma vdA=\int \gamma vrdrdzd\theta \mathrm{\backslash ]}$$ (18)

According to the finite element shape function, the Jacobian function can be expressed as,


$$\mathrm{\backslash [}\left\{ \begin{array}{c} v=\sum_{i=1}^{6}N_{i}v_{i}\\ r=\sum_{i=1}^{3}L_{i}r_{i} \end{array}\right.\Rightarrow drdz=2AdL_{1}dL_{2}\mathrm{\backslash ]}$$ (19)

The power dissipation $P_{t}$ can be calculated by,


$$\mathrm{\backslash [}\begin{array}{c} P_{t}=4\pi A\gamma \int \sum_{i=1}^{6}N_{i}v_{i}\sum_{i=1}^{3}L_{i}r_{i}dL_{1}dL_{2}\\ =4\pi A\gamma \left[\begin{array}{c} \frac{v_{1}}{15}\left(\frac{r_{1}}{4}-\frac{r_{2}}{8}-\frac{r_{3}}{8}\right)+\frac{v_{2}}{15}\left(-\frac{r_{1}}{8}+\frac{r_{2}}{4}-\frac{r_{3}}{8}\right)+\frac{v_{3}}{15}\left(-\frac{r_{1}}{8}-\frac{r_{2}}{8}+\frac{r_{3}}{4}\right)+\\ \frac{v_{4}}{15}\left(r_{1}+r_{2}+\frac{r_{3}}{2}\right)+\frac{v_{5}}{15}\left(\frac{r_{1}}{2}+r_{2}+r_{3}\right)+\frac{v_{6}}{15}\left(r_{1}+\frac{r_{2}}{2}+r_{3}\right) \end{array}\right] \end{array}\mathrm{\backslash ]}$$ (20)

The power dissipation due to the plastic deformation with continuous quadratic velocity field is defined as


$$\mathrm{\backslash [}P_{e}=\int_{A}d_{p}dA=\int_{A}d_{p}rdrdzd\theta \mathrm{\backslash ]}$$ (21)

The same approach can be used to deal with the integral.


$$\mathrm{\backslash [}\begin{array}{c} P_{e}=(2r_{1}+r_{2}+r_{3})\left(k_{1}y_{1,1}^{1}+k_{2}y_{2,1}^{1}+k_{3}y_{3,1}^{1}\right)+(r_{1}+2r_{2}+r_{3})\left(k_{1}y_{1,2}^{1}+k_{2}y_{2,2}^{1}+k_{3}y_{3,2}^{1}\right)\\ +(r_{1}+r_{2}+2r_{3})\left(k_{1}y_{1,3}^{1}+k_{2}y_{2,3}^{1}+k_{3}y_{3,3}^{1}\right) \end{array}\mathrm{\backslash ]}$$ (22)

Where,


$$\mathrm{\backslash [}k_{1}=\frac{\pi c\cos\varphi A}{3},k_{2}=\frac{2\pi c\cos\varphi A}{3\left(1+\sin\varphi \right)},k_{3}=\frac{2\pi c\cos\varphi A}{3\left(1-\sin\varphi \right)}\mathrm{\backslash ]}$$ (23)

## Supporting information

S1 Fig Quadratic velocity elements. (TIF)

S2 Fig General layout and typical mesh of the problem (rough interface) for UB method. (TIF)

S3 Fig The impact of the number of iterations on the load carrying capacity factor and the element count. (TIF)

S4 Fig Mesh refinement in the calculation of N_c_ for smooth interface with φ = 30°. (TIF)

S5 Fig Mesh refinement in the calculation of N_c_ for rough interface with φ = 30°. (TIF)
