# Supplementary figures and images for: Axisymmetric adaptive upper-bound finite element limit analysis formulation based on second-order cone programming for bearing capacity of circular footing
Source: PLoS One. 2025 Jun 5;20(6):e0321451. doi: 10.1371/journal.pone.0321451 (PMC12180546; doi:10.1371/journal.pone.0321451)

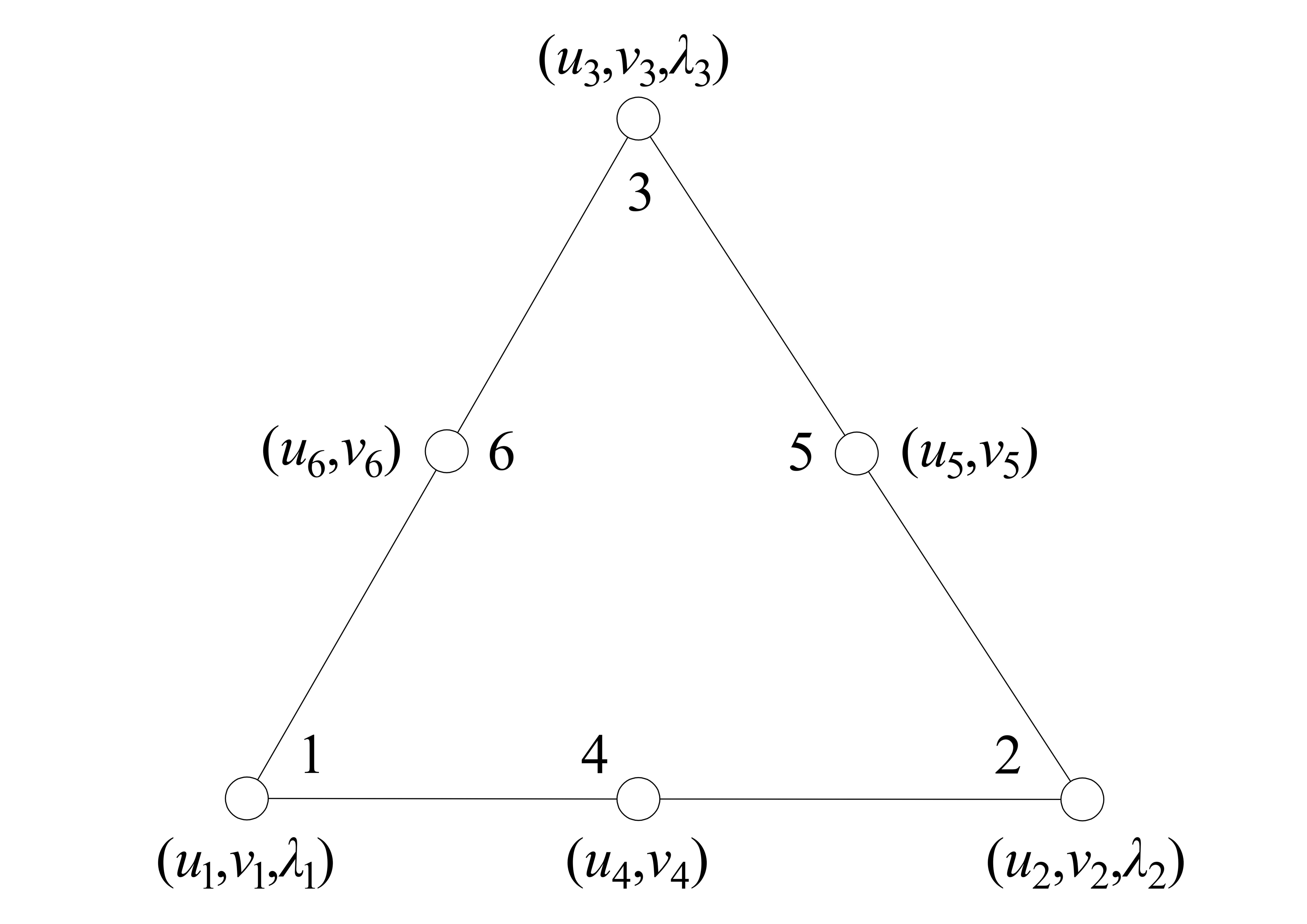

Supplement: S1 Fig — (TIF) [file pone.0321451.s001.tif]

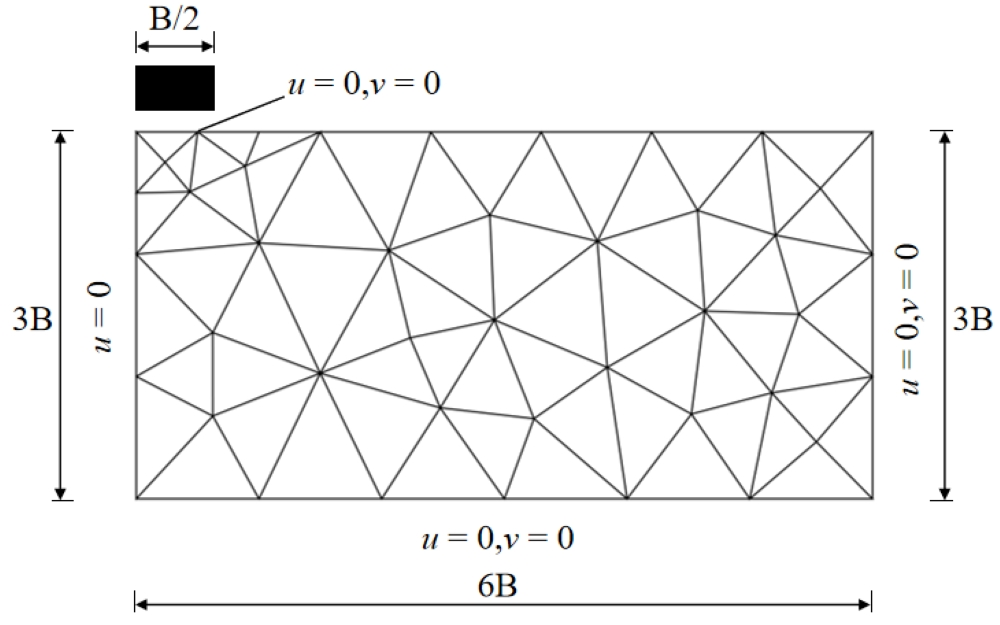

Supplement: S2 Fig — (TIF) [file pone.0321451.s002.tif]

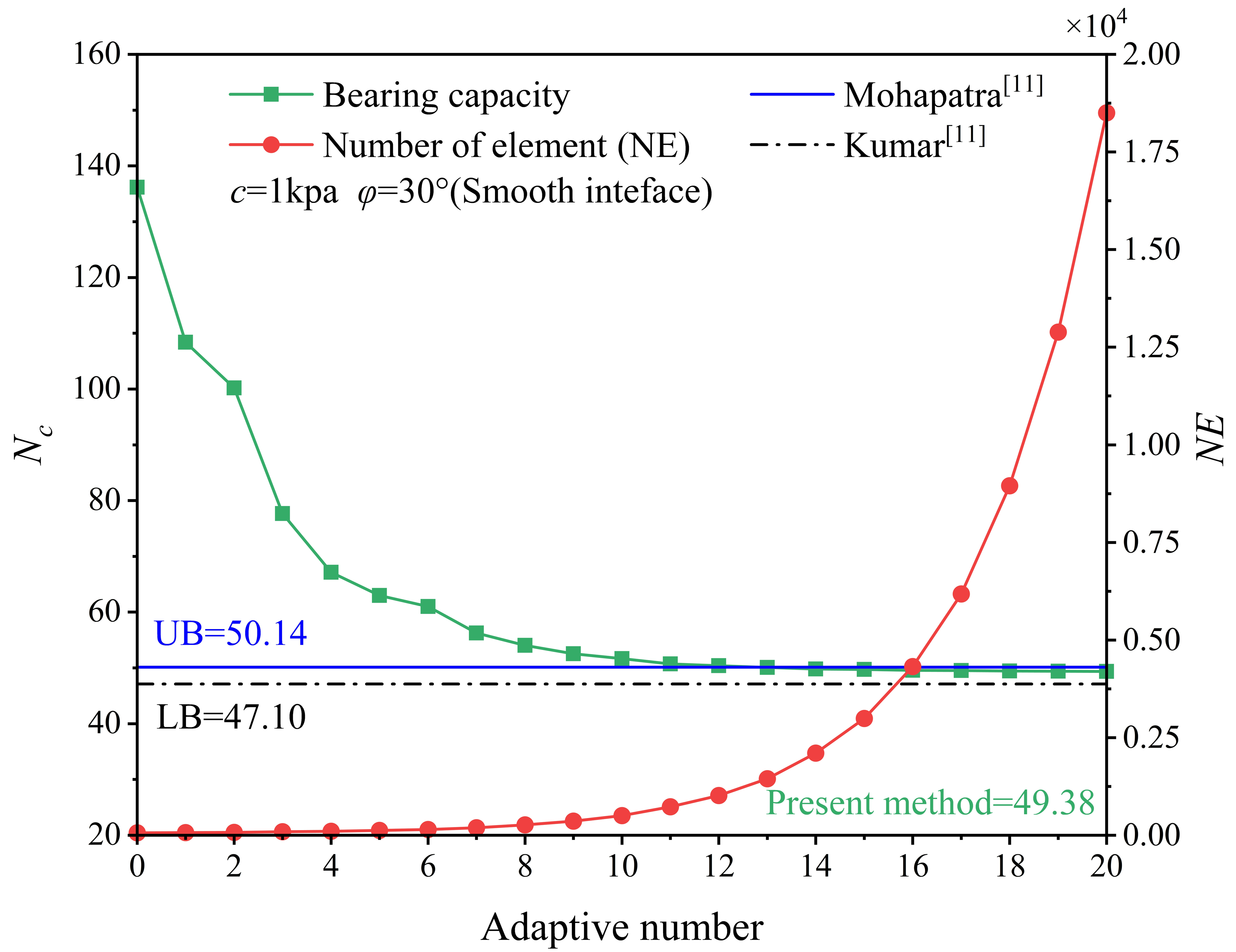

Supplement: S3 Fig — (TIF) [file pone.0321451.s003.tif]

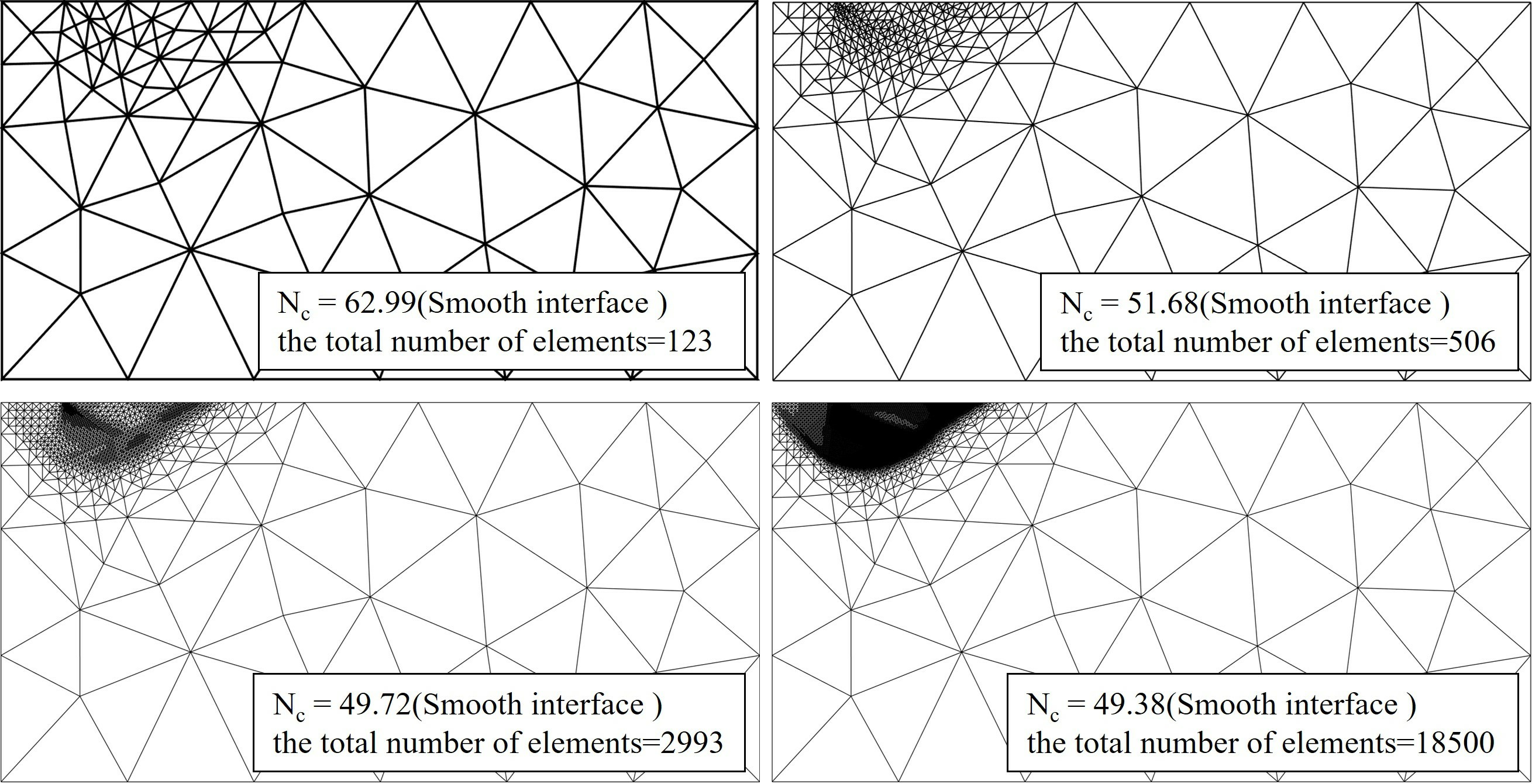

Supplement: S4 Fig — (TIF) [file pone.0321451.s004.tif]

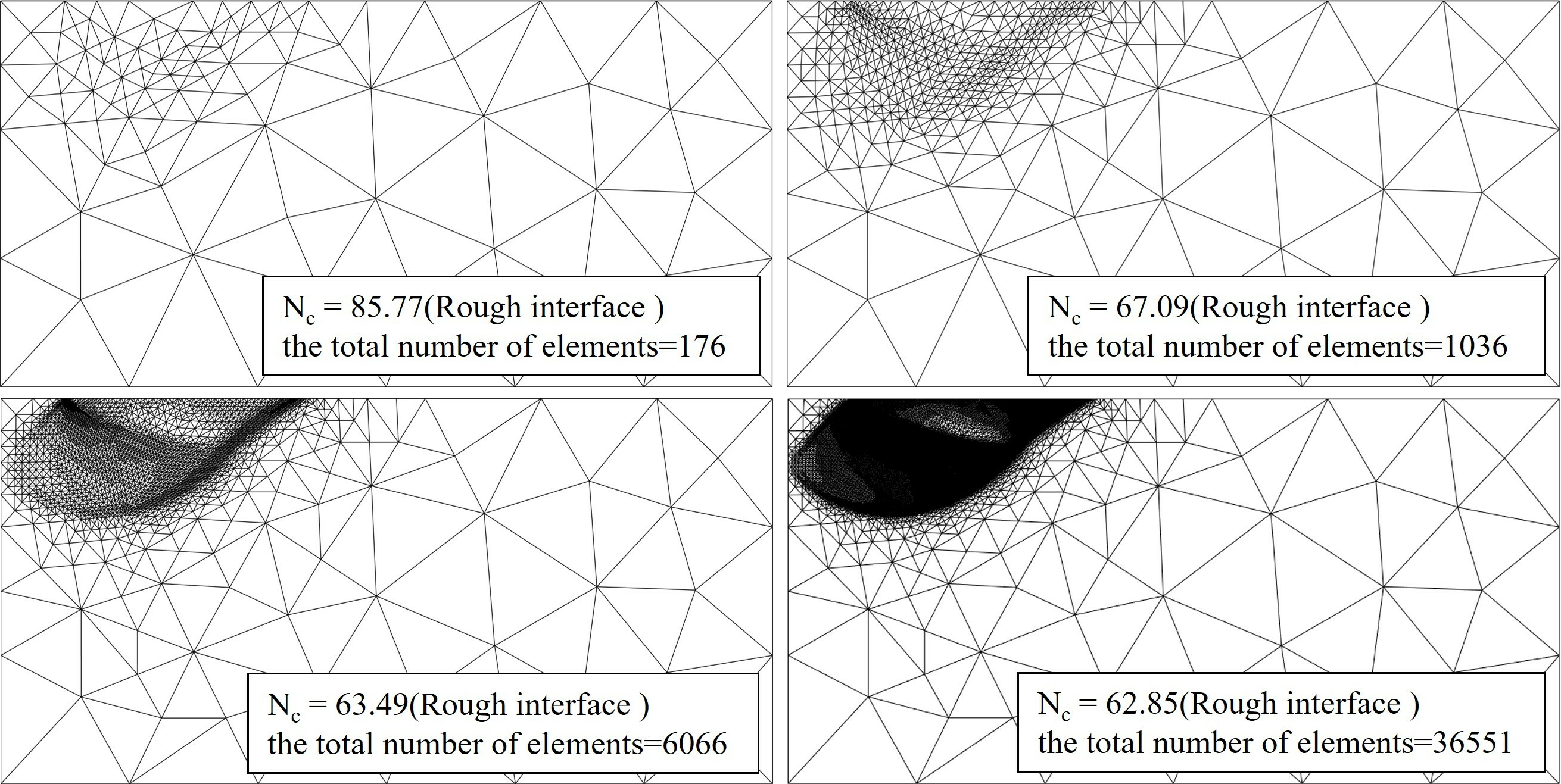

Supplement: S5 Fig — (TIF) [file pone.0321451.s005.tif]
